# Brief Comment on “The Role of Ventricular Arrhythmias Inducibility in Arrhythmic Risk Stratification in Arrhythmogenic Right Ventricular Cardiomyopathy: A Meta‐Analysis of Observational Studies”

**DOI:** 10.1002/joa3.70254

**Published:** 2025-12-09

**Authors:** Sidra Afzal, Nimra Afzal

**Affiliations:** ^1^ Fatima Jinnah Medical University Lahore Pakistan; ^2^ Anglia Ruskin University Cambridge UK

**Keywords:** cardiac death risk stratification, programmed ventricular stimulation, risk prediction, ventricular arrhythmias

Dear Editor,

I read with keen interest the recent meta‐analysis by Bazoukis G, Saplaouras A, Pillai N, Efthymiou P, Lee S, Sfairopoulos D, et al. analyzing the role of ventricular arrhythmias (VA) inducibility in arrhythmic risk stratification in patients with arrhythmogenic right ventricular cardiomyopathy (ARVC) [1]. All authors are to be acknowledged for summarizing data from different observational studies to shed light on a clinically faced key challenge. This letter focuses on some considerations about methodology and interpretation, which may provide assistance to clinicians in applying these outcomes strategically.

Firstly, while the meta‐analysis aims to provide pooled estimates of studies, the heterogeneity of the included studies requires scrutiny. Different factors such as study design, population, sites of stimulation, inducibility criteria, and different ventricular arrhythmia inducibility protocols played an important role in this variability. Such variations can influence the rate of arrhythmia detection and may restrict the applicability of pooled end results. Acknowledgment of this variability is crucial for the interpretation of risk stratification on the basis of inducibility.

Secondly, the studies that were included in the meta‐analysis were observational, increasing susceptibility to reporting and selection biases. For example, patients being subjected to electrical stimulation could have higher baseline risk profiles leading to a greater association between arrhythmic events and inducibility. Clinicians should reflect on all these confounding factors when projecting outcomes to wider ARVC populations.

Thirdly, the applicability of VA inducibility for risk prediction remained subtle. While VA inducibility can help in detecting higher‐risk patients, it should supplement rather than substitute comprehensive risk assessment comprising diagnostic imaging, genetic assessment, and standard clinical parameters. Different factors such as right ventricular dysfunction, male gender, age, fragmentation of the QRS complex, previous history of ventricular tachyarrhythmia, and syncope on initial evaluation are important predictors of arrhythmic events [2]. Programmed ventricular stimulation offers predictive information only when integrated with a risk calculator [3]. Integration of these modalities reinforces a comprehensive approach towards sudden cardiac death risk stratification in ARVC.

Lastly, this meta‐analysis highlights the importance of multicenter prospective studies with standard protocols. Such studies might offer more reliable estimates of the VA inducibility predictive value and assist in the development of guidelines for the management of ARVC.

To conclude, authors offered a significant synthesis of recent evidence on VA inducibility in ARVC. Prudent reflection on observational biases, heterogeneity, and incorporation with comprehensive clinical assessment is crucial for implementing these outcomes practically.

## Conflicts of Interest

The authors declare no conflicts of interest.
